# Data on UPLC/MS method validation for the biodegradation of pharmaceuticals and intermediates by a fungal consortium and on T47DK-Bluc reporter gene assay to assess the reduction of their estrogenic activity

**DOI:** 10.1016/j.dib.2019.104336

**Published:** 2019-07-30

**Authors:** Teddy Kabeya Kasonga, Martie A.A. Coetzee, Catherina Van Zijl, Maggy Ndombo Benteke Momba

**Affiliations:** a Department of Environmental, Water and Earth Sciences, Faculty of Sciences, Tshwane, University of Technology, P/B X 680, Pretoria, 0001, South Africa; b Department of Urology, University of Pretoria, Private Bag X323, Arcadia, 0007, Pretoria, South Africa

**Keywords:** Agonist E_2_, Antagonist ICI 182,780, UPLC/MS method, T47D-KBluc bioassay

## Abstract

In term of pharmaceutical and their intermediate compounds analysis, UPLC/MS method is a valuable equipment to achieve better confirmation on their biodegradation by fungi. The T47D-KBluc reporter gene assay is an appropriate tool to investigate to removal of estrogenic and antiestrogenic activities of pharmaceuticals and their metabolites from a synthetic wastewater. A consortium of isolated South African indigenous fungi *Aspergillus niger*, *Mucor circinelloides*, *Trichoderma longibrachiatum*, *Trametes polyzona* and *Rhizopus microspores* was found to perform a removal of pharmaceuticals and their metabolites and to reduce their estrogenic activity below the limit of detection in a sequencing batch reactor. Here are presented data regarding the phenolic compounds list and the method validation for UPLC/MS analysis used for selected pharmaceutical compounds namely carbamazepine, diclofenac, ibuprofen and their metabolites, as well as the T47D-KBluc bioassay using as positive control, the agonist E_2_ for estrogenic activity and the antagonist ICI 182,780 for antiestrogenic activity. For better understanding of the data presented in this paper, please see the research paper “Removal of pharmaceutical’ estrogenic activity of sequencing batch reactor effluents assessed in the T47DK-Bluc reporter gene assay” [1].

**Table:** Specifications Table

Subject area	*Microbiology, Chemistry, Environmental science*
More specific subject area	*Bioremediation of pharmaceutical wastewater by fungi*
Type of data	*List, tables, figures.*
How data was acquired	*Ultra-Performance Liquid Chromatography Water Acquity UPLC® system hyphenated to a quadrupole-time-of-flight (QToF) instrument operating with MassLynx™ (version 4.1) software (Waters Inc., Milford, Massachusetts, USA) was used for data acquisition and processing. Estrogenic and antiestrogenic activity was calculated from relative light unit using LUMIstar OPTIMA luminometer (BMG Labtech, Germany)*
Data format	*Raw and Analysed*
Experimental factor	*Solid phase extraction (SPE) method using Supel^TM^ Select Supelco HLB 500 mg, 12 mL plastic cartridges (Sigma Aldrich, South Africa) was performed for UPLC/MS samples loaded at pH 2.5, eluted with methanol (HPLC grade, Sigma Aldrich, South Africa). SPE Oasis® HLB 60 μm (LP), 500 cc glass cartridges (Water Microsep Pty Ltd, South Africa) were used for T47D-KBluc's samples. Samples were loaded at pH 3 and eluted using ethanol (HPLC grade, Sigma Aldrich, South Africa).*
Experimental features	*Synthetic wastewater samples containing pharmaceuticals (PhCs) were collected from a sequencing batch reactor driven by a fungal consortium and run at the retention time of one day. Estrogenic activity of PhCs and their metabolites was investigated using T47D-KBluc breast cancer cells according to the method developed by the United States Environmental Protection Agency.*
Data source location	*University of Pretoria, Pretoria, South Africa.*
Data accessibility Related research article	*All data are presented in this paper*. *T.K. Kasonga, M.A.A. Coetzee, C. Van Zijl, M.N.B. Momba, Removal of pharmaceutical estrogenic activity of sequencing batch reactor effluents assessed in the T47D-KBluc reporter gene assay. J. Environ. Manage. 240 (2019) 209–218*.

**Table:** 

**Value of the data** • The data highlight characteristics of positive controls 17-β estradiol (E2) and ICI 182,780 used as positive control in the T47D-KBluc bioassay and their scientific names according to the International Union of Pure and Applied Chemistry (UIPAC). • The data show a list of the potential estrogenic active phenolic compounds displaying endocrine-disrupting properties in the environment, because of their complex structures and masses normally ranging from 200 to 1000 Da. • The data provide details in the development and validation of the UPLC/MS method used for the analysis of pharmaceutical compounds and their transformation fragment ions referred as metabolites, for better understanding of the results in the research paper. • A protocol is provided with details for the preparation of media, general cell culture procedure of T47D-KBluc breast cancer cells, to be used for further insights. • The data display the T47D-KBluc method validation in terms of the calibration curves of E2 and ICI 182,780 using Graphpad Prism Software (version 4).

## 1 Data

**Fig. 1:**
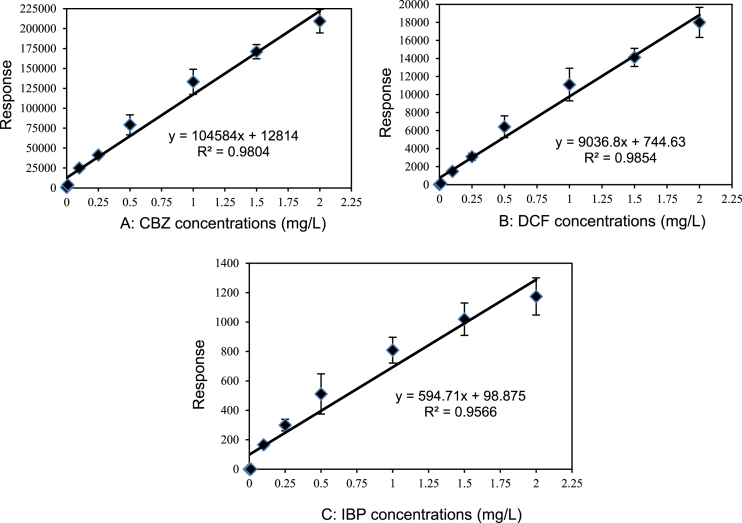
Pharmaceutical calibration curves (CBZ, DCF and IBP).

**Fig. 2:**
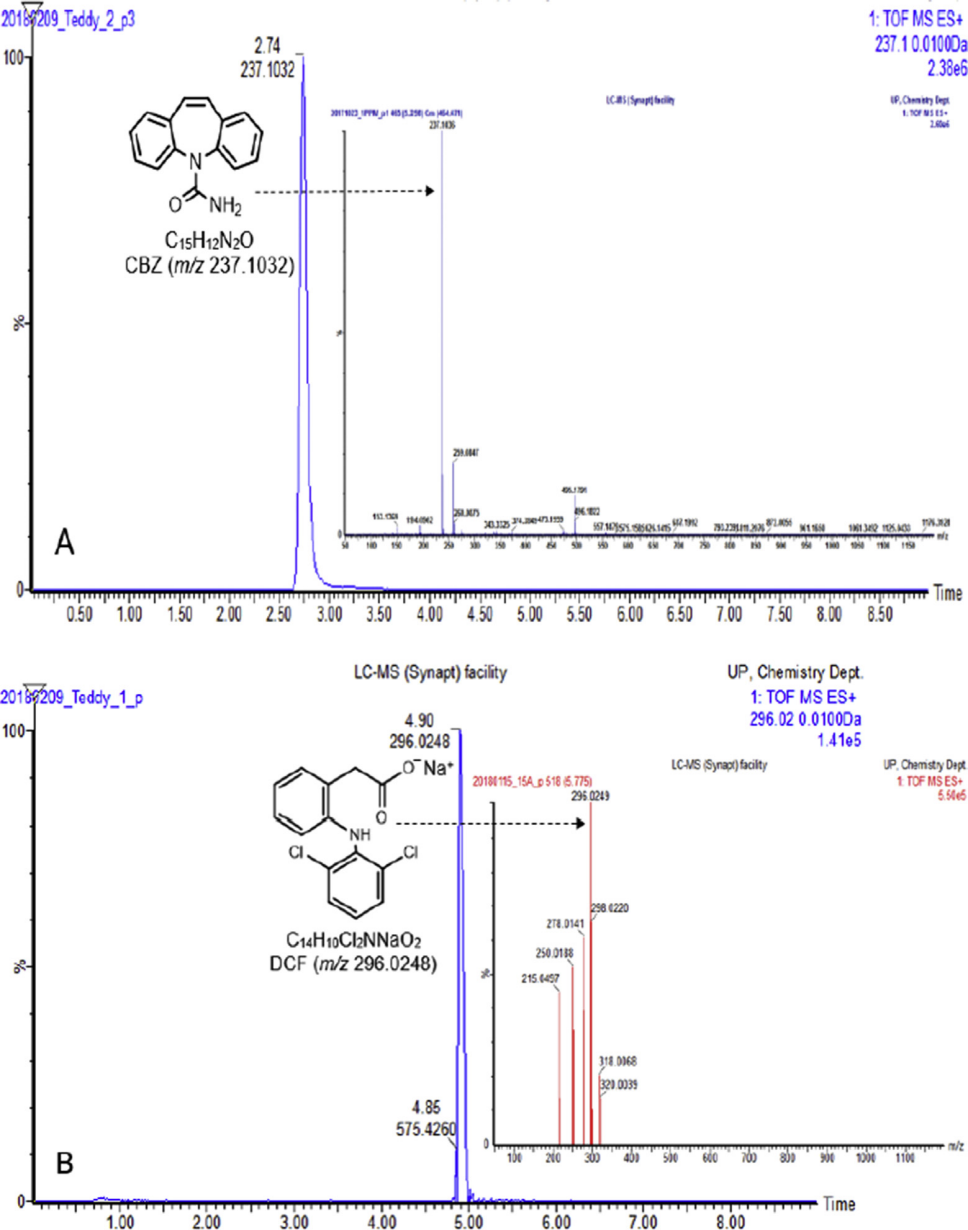

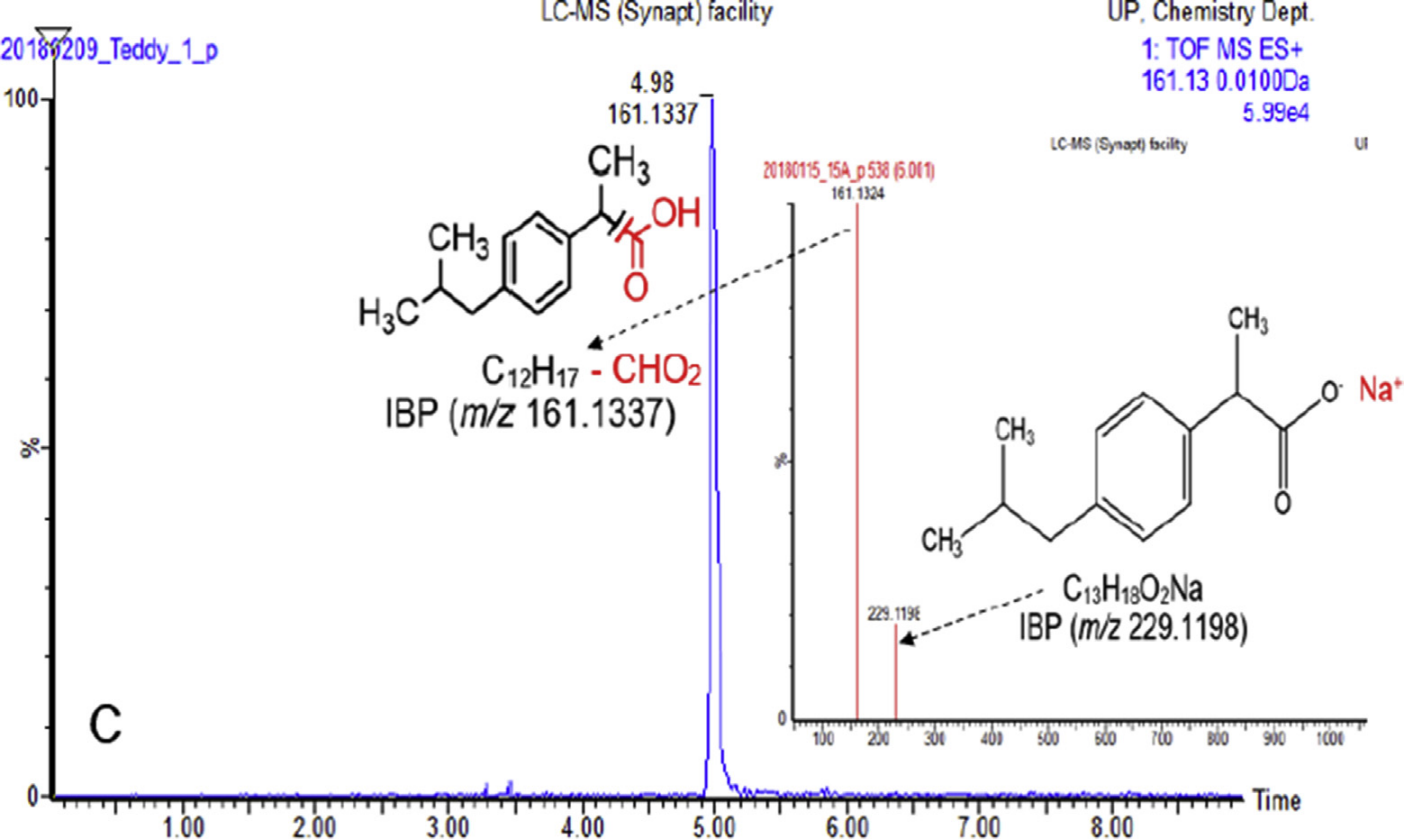
A: Selected UPLC-(+)-ESI-QToF-MS chromatogram and production spectrum of CBZ. B: Selected UPLC-(+)-ESI-QToF-MS chromatogram and production spectrum of DCF. C: Selected UPLC-(+)-ESI-QToF-MS chromatogram and production spectrum of IBP.

**Fig. 3:**
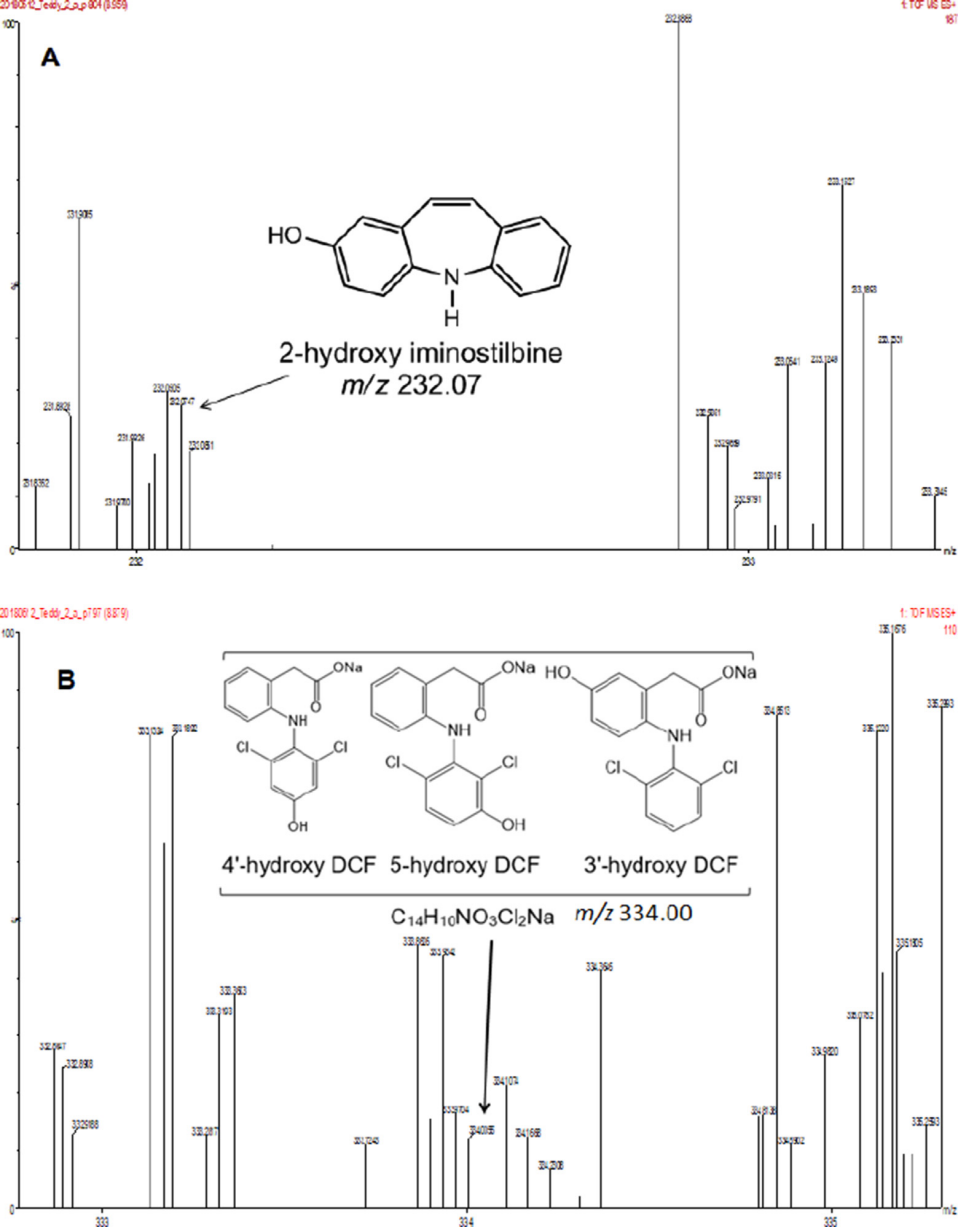

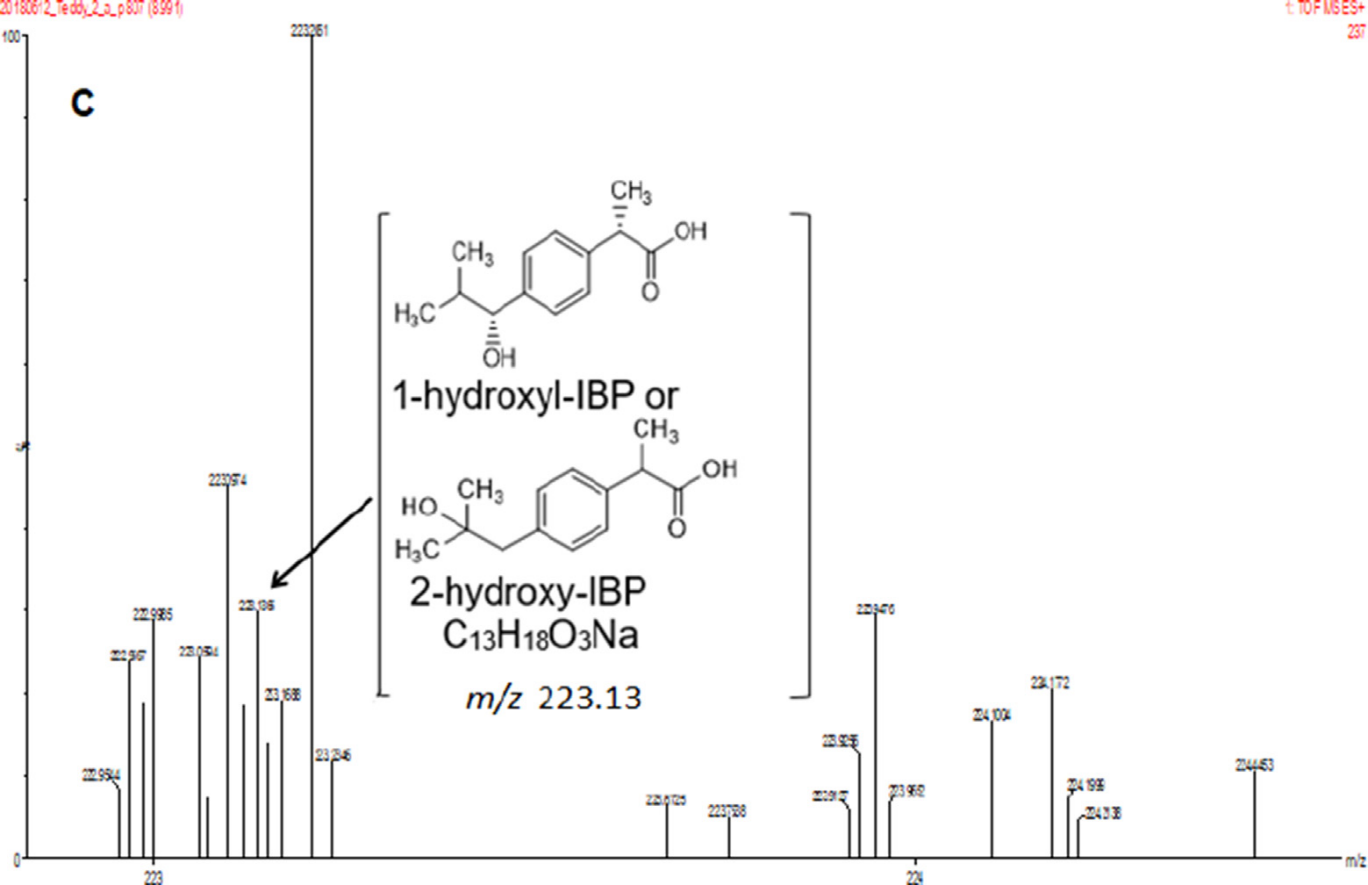
A: Few UPLC-(+)-ESI-QToF-MS production spectra of CBZ biodegradation products from synthetic wastewater in the SRB driven by a fungal consortium of the indigenous isolate fungi. B: Few UPLC-(+)-ESI-QToF-MS production spectra of DCF biodegradation products from synthetic wastewater in the SRB driven by a fungal consortium of the indigenous isolate fungi. C: Few UPLC-(+)-ESI-QToF-MS production spectra of IBP biodegradation products from synthetic wastewater in the SRB driven by a fungal consortium of the indigenous isolate fungi.

**Fig. 4:**
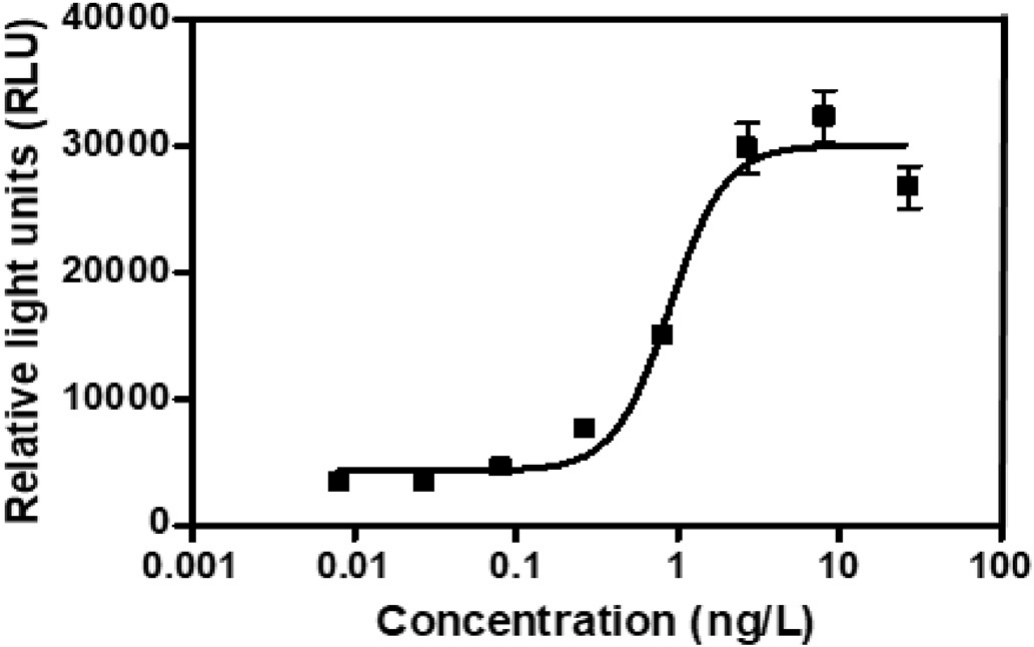
The 17-Beta estradiol (E_2_) calibration curve for estrogenic activity assay.

**Fig. 5:**
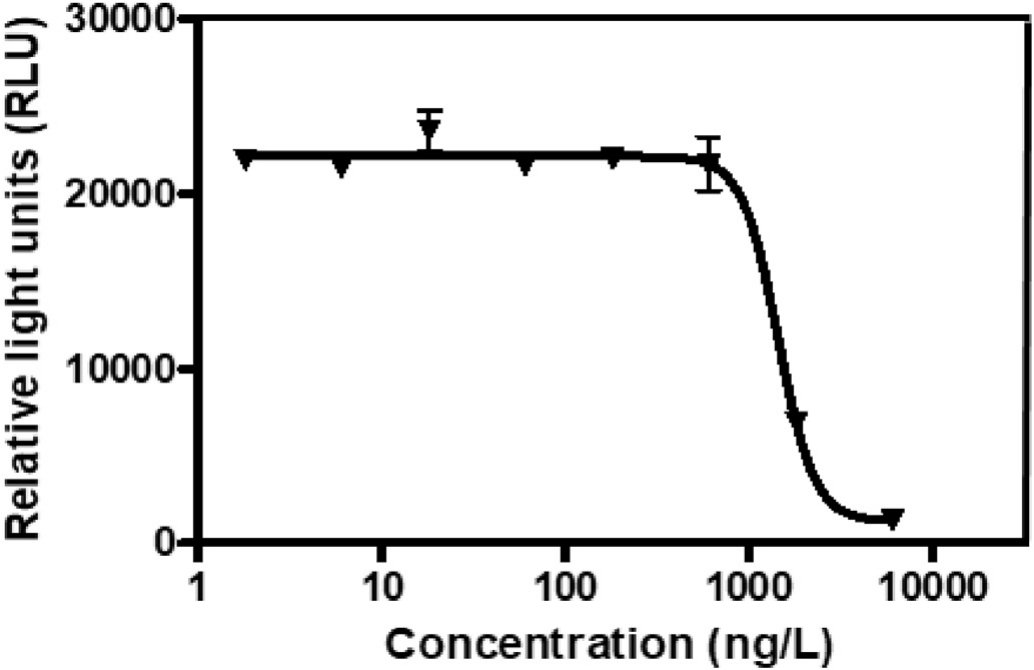
The ICI 182,780 calibration curve for the anti-estrogenic activity assay.

In the present report, authors present data on the method development and validation to confirm the removal of pharmaceutical compounds (PhCs) and their metabolites, especially the reduction of the metabolite’ estrogenic activity in a sequencing batch reactor (SBR) [1], as it has been reported that intermediate products might be more toxic than the initial parent compounds [2]. The UPLC-MS method development parameters are shown in Table 1 with the chromatogram and production spectrum in Fig. 2 for the selected PhCs, namely carbamazepine (CBZ), diclofenac (DCF) and ibuprofen (IBP). The SPE-UPLC/MS method was validated (Table 2) in term of matrix effect (Equation (1)), linearity of calibration curve (Fig. 1), sensitivity, accuracy and precision, specificity and selectivity [3], [4]. Fig. 2 displays the UPLC-chromatogram and MS-spectrum of selected parent PhCs [CBZ at *m/z* 237.10 (Fig. 2A), DCF at *m/z* 296.02 (Fig. 2B) and IBP an *m/z* 161.13 and 229.11 (Fig. 2C)], while Fig. 3 gives some intermediate spectrum (at *m/z* 232.07, 334.00 and 223.13). A consortium of South African indigenous fungi *Aspergillus niger*, *Mucor circinelloides*, *Trichoderma longibrachiatum*, *Trametes polyzona* and *Rhizopus microspores* previously isolated and identified was found to be capable to remove the selected PhCs, namely CBZ, DCF and IBP in the SBR run at a retention time of one day. Pharmaceutical compound metabolites have exhibited higher estrogenic activity assayed in the T47D-KBluc (EEq 2.69 ± 0.17 ng/L) [1]. In the T47D-KBluc gene reporter bioassay, to determine the EEq values using Graphpad Prism software (version 4), X-values (concentrations) were log transformed and the E_2_ and ICI 182,780 curves were fitted (sigmoidal function, variable slope). Y-values and were corrected in order to correspond to dilution factors and determine their EEq values [1], [5]. The T47D-KBluc standard curves are shown in Fig. 4, Fig. 5.

**Table 1:** Method development parameters.

Analytes	Therapeutic class	m/z	Rt/min	Linear-range mg/L	Noise start	Noise end
CBZ	anti-epileptic	237.10	2.750 ± 0.1	0.001–2	2.5	2.6
DCF	anti-analgesic	296.02	4.903 ± 0.2	0.001–2	4.1	4.5
IBP	anti-analgesic	229.12	4.995 ± 0.3	0.001–2	4.5	4.6

**Table 2:** Method validation parameters.

Analytes	Experimental r^2^	Calculated r^2^	LoD (mg/L)	LoQ (mg/L)	Mean recovery (%)	RSD (%)
CBZ	0.969	0.980	9.71 × 10^−5^	3.24 × 10^−4^	106.20	8.45
DCF	0.965	0.985	2.4 × 10^−4^	8.1 × 10^−4^	104.62	7.33
IBP	0.927	0.957	4.47 × 10^−3^	17.16 × 10^−3^	102.89	8.50

## 2 Experimental design, materials and methods

### 2.1 Agonist E_2_ and antagonist ICI 182,780

The T47D-KBluc reporter gene assay method was developed by the United States Environmental Protection Agency (USEPA) to screen chemical compounds and environmental samples for estrogenic and anti-estrogenic activities. T47D human breast cancer cells, which naturally express both human estrogen receptor (ER)-α and -β, were transferred with an estrogen-responsive element luciferase reporter gene construct [5]. The 17-β estradiol (E_2_) agonist positive control is a natural steroid sex hormone produced by the ovaries named according to UIPAC as (8R,9S, 13S, 14S, 17S)-13-methyl-6,7,8,9,11,12,14,15,16,17-decahydrocyclopenta[a]phenanthrene-3,17-diol [1], with molecular mass of 272.388 g/mol. The antagonist ICI 182,780 is a synthetic steroidal antiestrogen derived from E_2_ named by IUPAC as (7R, 8R, 9S, 13S, 14S, 17S)-13-methyl-7-[9-(4,4,5,5,5-pentafluoropentylsulfinyl)nonyl]-6,7,8,9,11,12,14,15, 16,17-decahydrocyclopenta[a]phenanthrene-3,17-diol [1], with a molecular mass of 606.777 g/mol.

### 2.2 List of phenolic compounds

Phenolic compounds include simple phenols, tyrosine derivatives, phenolic acids/phenolic aldehydes, acetophenones, phenylacetic acids, hydroxycinnamic acids, bisphenols, phenylpropenes, benzophenones, coumarins, naphthoquinones, stilbenes/stilbenoids, anthraquinones, chalcones/chalconoids, flavolans, flavones/flavonoids, diarylheptanoids and hydroxylated polycyclic aromatic hydrocarbons. In addition to these, chemicals without a phenolic ring were also found to be exhibiting estrogenic activity, these comprise anilines, perfluorinated compounds, carboranes, indoles, phthalates and terpenes/terpenoids. Among the terpene group there are compounds such as monoterpenes, diterpenes, triterpenes, tetraterpenes, sesquiterpenes, steroids, sterols, saponins and meroterpenes [6].

### 2.3 UPLC/MS method development and validation

The Ultra-Performance Liquid Chromatography tandem Mass Spectrometry (UPLC/MS) method was used for the present data. Pharmaceuticals separation and their detection were carried out using a Waters® Synapt G2 high definition mass spectrometry system (Waters Inc., Milford, Massachusetts, USA). The system consisted of a Waters Acquity UPLC® system hyphenated to a quadrupole-time-of-flight (QToF) instrument operating with MassLynx™ (version 4.1) software (Waters Inc., Milford, Massachusetts, USA) for data acquisition and processing. The internal control was used to compensate for instrumental drift, ensuring good mass accuracy, throughout the duration of the runs. The instrument was calibrated using sodium formate clusters and Intellistart functionality (mass range 112.936 - 1 132.688 Da). Resolution of 20,000 at *m/z* 200 [full width at half maximum (FWHM)] and mass error within 5 mDa were obtained. The source conditions were as follows: the capillary voltage for ESI was 2.6 kV for positive mode ionisation. The source temperature was set at 120 °C, the sampling cone voltage at 25 V, extraction cone voltage at 4.0 V and cone gas (nitrogen) flow at 10.0 L/Hr. The desolvation temperature was set at 350 °C with a gas (nitrogen) flow of 600.0 L/Hr. Quantitative data-independent acquisition (DIA) was done using two simultaneous acquisition functions with low and high collision energy (MS^E^ approach) with a QTOF instrument. Fragmentation was performed using high energy collision induced dissociation (CID). The fragmentation energy was set at 2 V and 3 V for the trap and collision energy, respectively. The ramping was set from 3 to 4 V and 20–40 V for the trap and transfer collision energy, respectively.

Mass spectral scans were collected every 0.3 seconds. The raw data was collected in the form of a continuous profile. Mass to charge ratios (*m/z*) between 50 and 1 200 Da were recorded. Mobile phase and other parameters including the column used, flow rate and injection conditions are provided within the paper.

#### 2.3.1 Method development and validation

A control blank matrix extract with increments of known analyte concentrations of selected drugs (CBZ, DCF and IBP) was used for the method development. The blank matrix (ddH_2_O) was spiked with a mixture of pharmaceutical standard solutions (CBZ, DCF and IBP) at the concentration of 0.01, 1 and 5 mg/L. According to their monoisotopic mass, all targeted drugs were quantified in positive ion mode (ESI+) while their detection in low energy was performed in a reaction monitoring mode of respectively *m/z* 237.10 (CBZ), *m/z* 296.02 (DCF) and *m/z* 229.12 (IBP for IBP + Na). The following Table 1 showed the method development parameters set to perform the UPLC/MS.

Considering the endogenous compounds from the aerated batch flasks (including fungal enzymes and other compounds), the ionisation of the targeted analytes could be increased or suppressed. The effect of the fungal medium constituents as a matrix effect was evaluated by comparing the slope of the selected pharmaceutical calibration curves in spiked medium extracts and in spiked pure solvent [7]. The matrix effect using SPE HLB column extracted was evaluated and the values less than 20% were considered acceptable [8]. The procedure described by Kasonga et al. (2019) was used. The following Equation (1) was used to determine the matrix effect (*ME*) in the working conditions.

(1) $$ME=\frac{Slope\;of\;fungal\;medium-matched\;calibration\;curve}{Slope\;of\;the\;s\;\tan\;dard\;calibration\;curve}x100$$

The method was thereafter validated in terms of required parameters including: linearity of the calibration curve, specificity and selectivity, sensitivity, precision and accuracy, and recovery [9].

#### 2.3.2 Linearity of calibration curve

Calibration curves (Fig. 1) were carried out by plotting the peak area ratios (or signal response) of each analyte versus the theoretical eight (8) point concentrations of the spiked drug in methanol-water (1:1, v/v). The mixture CBZ, DCF and IBP at individual concentrations of 0.001, 0.01, 0.1, 0.25, 0.5, 1, 1.5 and 2 mg/L were prepared in triplicate of each concentration from filtered fungal batch media. The filtered medium without selected pharmaceuticals was used as a blank. Prior to the injection in UPLC/MS, the SPE was performed for all blank and analyte media. The linearity of the calibration curves was assessed throughout the recorded *r*^2^ values and equations given by the instrument. The recorded values were found to be in the acceptable range >0.9 as suggested in literature [3], [10].

#### 2.3.3 Sensitivity

The sensitivity of the method was evaluated in terms of the limit of detection (LoD) and the limit of quantification (LoQ) for each selected pharmaceutical and was assessed during the determination of the linear range of the calibration standards as those concentrations giving a signal to noise ratio (S/N) of 3 and 10 respectively. The LoD and the LoQ ware calculated according to literature (Gracia-Lor et al., 2010). The following Equations (2), (3) were used to determine the LoD and LoQ respectively.

(2) $$\mathrm{LoD}\;=\frac{\mathrm{lowest}\;\mathrm{concentration}\;\mathrm{of}\;\mathrm{standard}}{\mathrm{instrumental}\;S/N\;\mathrm{for}\;\mathrm{lowest}\;\mathrm{concentration}\;\mathrm{of}\;\mathrm{standard}}x\;3$$

(3) $$\mathrm{LoQ}\;=\frac{\mathrm{lowest}\;\mathrm{concentration}\;\mathrm{of}\;\mathrm{standard}}{\mathrm{instrumental}\;S/N\;\mathrm{for}\;\mathrm{lowest}\;\mathrm{concentration}\;\mathrm{of}\;\mathrm{standard}}x\;10$$

#### 2.3.4 Accuracy and precision

In respect of assessing the reproducibility and measuring the closeness among the replicates, daily precision and accuracy, CBZ, DCF and IBP (mixed working standard solution) at low (0.01 mg/L and 0.1 mg/mL), middle (0.75 mg/L and 1 mg/L), and high (2 mg/L) concentrations were spiked in triplicates (*n* = 3) in accordance with the method proposed by previous investigators [4], [11]. The daily precision (intra-day/inter-day assay), one spike per day over three days, was then assessed by determining the method accuracy (expressed as % recovery) and precision [expressed as repeatability in terms of % relative standard deviation (RSD)]. The criteria for the tolerability were that the precision level should not exceed 15%–20% and the averaged assessment of the accuracy should be within ±15–20% (Wang et al., 2011). The % Recovery and % RSD of the method were determined using Equations (4), (5) respectively.

(4) $$\%\;\mathrm{Recovery}=\frac{\mathrm{drug}\;\mathrm{experimentally}\;\mathrm{determined}\;\mathrm{concentration}}{\mathrm{true}\;\mathrm{spike}\;\mathrm{concentration}}x\;100$$

(5) $$\%\;\mathrm{RSD}\;=\frac{\mathrm{standard}\;\mathrm{deviation}}{\mathrm{mean}}\;x\;100$$

#### 2.3.5 Specificity and selectivity

These parameters were evaluated in terms of chromatographic interferences from endogenous compounds which were eliminated by comparing the chromatograms of the blank medium and the analyte spiked medium (CBZ, DCF and IBP, Table 2) as described by previous investigators [10], [11]. The analyte samples at the concentrations of 0.001, 1 and 5 mg/L were used for the data.

### 2.4 UPLC-(+)-ESI-QToF-MS chromatogram and production spectrum of selected PhCs

The Fig. 2 displayed UPLC-chromatogram and MS-spectrum of selected parent PhCs, namely CBZ at *m/z* 237.10, DCF at *m/z* 296.02 and IBP fragment ion at *m/z* 161.13 and its sodium adduct at *m/z* 229.12. Fig. 3 demonstrated peaks of illustrative spectrum of transformation fragment ions from parent PhCs.

### 2.5 T47D-KBluc general cell culture production and bioassay

#### 2.5.1 Bioassay media preparation

Media preparation and the assay procedure were performed according to the standard assay procedures [5], [12], [13]. Media components were prepared in double distilled water (ddH_2_O) from a Milli-Q synthesis ultrapure water system (Merck, Germany), equipped with an EDS-Pak Polisher (an activated carbon-based filter) to produce EDC-free water. Briefly explained, the RPMI medium (medium formulation developed at Roswell Park Memorial Institute) was prepared by dissolving in 1L ddH_2_O: 1 bottle of RPMI-1640 powder (Sigma Aldrich, USA), 2.5 g of D-(+)-glucose (Merck, Germany), 1.5 g of sodium bicarbonate (NaHCO_3_, Sigma-Aldrich, USA), 10 mL of 4-(2-Hydroxyethyl)piperazine-1-ethanesulfonic acid buffer solution (1 M) (Gibco, Life Technologies corporation, UK) and 10 mL of sodium pyruvate solution (100 mM) and the pH was adjusted to 7.3 (with 5 N, HCl). The medium was filter sterilised using a 0.22 μm bottle top filter and stored at 4 °C.

The maintenance medium consisted of RPMI medium supplemented with 10% foetal bovine serum, characterized, (FBS, Hyclone Laboratories, USA) and 100 U/mL penicillin, 100 U/mL streptomycin and 0.25 μg/mL amphotericin B (antibiotic/antimycotic solution, Sigma-Aldrich USA). The medium used to withdraw the cells from steroids one week before the bioassay, contained RPMI medium and 10% of c/d FBS (charcoal/dextran treated FBS, Hyclone Laboratories, USA), while the dosing medium was composed of RPMI medium with 5% of c/d FBS.

Hank's balanced salt solution (HBSS, Gibco Life Technologies corporation, UK) was prepared by diluting a 10x concentrated HBSS solution in sterile EDC-free ddH_2_O (sterilised by autoclaving for 20 min at 121 °C). The concentrated phosphate buffer saline (PBS, Gibco Life Technologies corporation, UK) and the reporter lysis buffer (Promega, USA) were diluted 10x and 5x in ddH_2_O, respectively. Trypsin (0.5% EDTA, Gibco Life Technologies Corporation, UK) was prepared by diluting 10x the concentrated solution in HBSS. Glycylglycine 1 M stock solution (pH 7.8) was prepared in ddH_2_O by dissolving the appropriate mass of glycylglycine (MW 132.1, Sigma-Aldrich, USA). Adenosine 5′-triphosphate (ATP, MW 551.1, Sigma-Aldrich, USA) 0.1 M stock solution and bovine serum albumin (BSA, Sigma-Aldrich, USA) 50 mg/mL were also made by dissolving an accurate mass in ddH_2_O. The reaction buffer solution (pH 7.8) consisted of 2 mL glycylglycine (1 M), 5 mL ATP (0.1 M), 1 mL BSA (50 g/L) and 1.5 mL MgCl_2_ (1 M) added to 90 mL of ddH_2_O. All the solutions were stored at 4 °C, except the concentrated trypsin and BSA stock solution aliquots, which were stored at −20 °C and the ATP stock solution aliquots at −80 °C.

A 1 mM solution of luciferin was prepared in ddH_2_O using beetle D-luciferin (potassium salt MW 318.41, Promega, USA) and stored in aliquots at −80 °C. The β-estradiol (E_2_, MW 272.39, Sigma-Aldrich, USA) 10 mM and ICI 182,780 (MW 606.77, Tocris biosciences, USA) 10 mM stock solutions were prepared in methanol (HPLC grade, Merck, Germany) and stored in wrapped foil vials at −20 °C.

#### 2.5.2 T47D-KBluc general cell culture procedures

The T47D-KBluc cells used for the bioassay were purchased from the American Type Culture Collection (ATCC, USA) and aseptic procedures were performed in a Type II bio-hazardous safety cabinet. Cell stock cultures were stored at −80 °C for 6–12 months and were thawed in a water bath at 37 °C. Cells were grown and maintain in RPMI medium (medium formulation developed at Roswell Park Memorial Institute, Sigma Aldrich, USA) with 10% foetal bovine serum, characterized, (FBS, Hyclone Laboratories, USA) and antibiotic/antimycotic solution using 25 cm^2^ or 75 cm^2^ tissue culture flasks and were incubated at 37 °C in a humidified 5%CO_2_ water-jacketed incubator (Nuaire™, USA). The cells were trypsinized and subcultured when confluent, at 3 to 4-day intervals. To trypsinize, the cells were rinsed 2x using 5 mL of Hank's balanced salt solution (HBSS, Gibco Life Technologies corporation, UK). The HBSS was then removed and 5 mL of trypsin was added to each flask. The flasks were incubated again for 3 min in the same conditions (37 °C and 5% CO_2_). The excess trypsin was discarded from the tissue culture flasks and the cells detached from the surface by gently tapping the flask against the hand. Cells were thereafter suspended in 10 mL of maintenance RPMI medium and divided 1/3 into subcultures.

#### 2.5.3 Standard curves of the bioassay for E_2_ and ICI 182,780

Fig. 4 displayed the E_2_ standard curve fitted using Graphpad Prism software (version 4). Fig. 5 demonstrated the anti-estrogenic activity of the assay was quantified from the fitted ICI 182,780 curve (co-incubated with 0.1 nM E_2_).

## Ethics approval

The human cells used in these data were not harvested by us. We used an established and modified cell line from the ATCC® CRL-2865™.
